# The impact of nursing students on the health-related quality of life and perceived social support of a rural population in Ecuador: effects of a service-based learning course

**DOI:** 10.1186/s12939-018-0734-z

**Published:** 2018-02-02

**Authors:** Rebecca L. Walcott, Angela M. Murcia, Gloria M. Berry, Christian F. Juna, María Isabel Roldós, Phaedra S. Corso

**Affiliations:** 1 Department of Health Policy and Management, The University of Georgia, 311D Wright Hall, 100 Foster Road, Athens, GA 30602 USA; 2 Faculty of Nursing, Pontificia Universidad Católica del Ecuador, Quito, Ecuador; 3 College of Health Sciences, Walden University, Minneapolis, MN USA

**Keywords:** Health-related quality of life, Nursing education, Public health nursing, Service-based learning, Community health, SF-12

## Abstract

**Background:**

Students seeking degrees in healthcare in Ecuador participate in community improvement projects and provide free health services under the supervision of faculty health professionals. The aim of this study is to determine the impact of a community-based intervention delivered by nursing students on health-related quality of life (HRQoL) and perceived social support of a rural population in Ecuador.

**Methods:**

A quasi-experimental non-equivalent control group design study was conducted in two rural communities in Tumbaco, Ecuador. Families from one rural community were invited to participate in the intervention, receiving 8 weekly home visits from nursing students. Families from a neighboring community were similarly recruited as wait-list controls. One member of each family was consented into the study; the final sample included 43 intervention participants and 55 control participants. HRQoL and perceived social support were assessed before and after the intervention in both groups. The SF-12 was used to measure HRQoL, including eight domain scores and two composite scores, and the Interpersonal Support Evaluation List was used as an indicator of perceived social support. Difference-in-differences (DD) analyses were conducted to mitigate the effects of any baseline differences in the non- equivalent control group design.

**Results:**

When compared to the control group, the intervention group realized significant improvements in the physical component summary score of the SF-12 (4.20, *p* < 0.05) and the physical function domain of the SF-12 (4.92, *p* < 0.05). There were no statistically significant differences for any other components of the SF-12 or in the measure of perceived social support.

**Conclusions:**

Nursing students completing their rural service rotation have the potential to improve the health-related quality of life of rural residents in Ecuador. Future research should continue to examine the impact of service-based learning on recipient populations.

## Background

Ecuador has experienced rapid economic development over the last few decades, but the benefits of development have not been equally distributed throughout the country. Approximately 36% of Ecuadorians live in rural areas, and nearly 35% of rural households live below the national poverty line (compared to only 16% of urban households) [1]. In addition, rural provinces display the highest rates of child malnutrition and illiteracy while also demonstrating the lowest rates of access to clean water and sanitation infrastructure [2]. It is estimated that 27% of all Ecuadorians do not have access to health services [3]. The unequal distribution of these social determinants of health is further exacerbated by an unequal distribution of health care providers. Ecuador reports approximately 20 physicians, 10 nurses, and 3 dentists per 10,000 Ecuadorians [4]; but the geographic disparity in number of physicians ranges from less than five to over 26 per 10,000 Ecuadorians [2].

In 1970, in an effort to address the gap in health determinants and outcomes between rural and urban populations, the government of Ecuador legislated one year of compulsory medical service for all new health professionals upon graduation (known as *medicatura rural* or *Año de Salud Rural*) [5]. Additionally, the more recent *Ley Orgánica de Educación Superior* (LOES) requires all students seeking higher education degrees to complete a community service internship or community improvement project [6]. As a result, the curriculum for health science degrees at many learning institutions includes service-based learning courses and internships with rural communities and vulnerable populations. Students seeking degrees in healthcare in Ecuador participate in community improvement projects and provide free health services under the supervision of faculty health professionals, thus conforming to the standards under LOES and to Ecuador’s National Plan for Wellbeing (*Plan Nacional del Buen Vivir*) [7]. For example, nursing students in the Family Nursing rotation at Pontificia Universidad Católica del Ecuador (PUCE) provide health education, increase access to health services, and develop family-specific plans of care to populations in rural communities near Quito, the country’s capital. The purpose of this service-based learning course is not only to meet the requirements of national legislation but also to improve the overall welfare and quality of life in these rural communities [8].

Although the rural health practice of students in service-based learning courses is believed to improve the quality of life in these communities, empirical evidence has yet to be established. Much research exists that assesses the educational and professional benefits of rural medical service for the provider [9–13]; however there is a startling lack of investigation on the effects of such programs on the recipient population. A handful of studies in high-income countries undertake a qualitative approach to describe the benefits of service-based learning for the recipient population, reporting that nursing students have the potential to effectively fill service gaps and can improve health-related behaviors in underserved populations [14–17]. One study quantitatively analyzed the impact of interprofessional teams of students, finding that a student-based home visitation program effectively increased preventive health measures among recipient households [18]. The results of these studies are encouraging; however, no studies have been conducted to-date that assess the impact of nursing student home visits on the health outcomes of a rural population in a low- or middle-income country.

The aim of this study was to determine the impact of the Family Nursing rotation of Pontificia Universidad Católica del Ecuador on health-related quality of life (HRQoL) and perceived social support of a rural population in Ecuador. HRQoL, while not a direct indicator of health, is a well-established proxy measure that reflects the impact of health status on overall quality of life [19]. Assessing an individual’s HRQoL gives insight into both physical and mental wellbeing, and changes in HRQoL over time can be used to describe the impact of personal experiences and/or health-related interventions [20–22]. Perceived social support is directly linked to quality of life and refers to an individual’s interpersonal network and the emotional, instrumental, and informational resources that network interactions provide [23, 24]. Assessments of individuals’ perceived social support over time can reveal changes in social function and interpersonal support, factors which can impact quality of life and disease severity [25–29]. For this study, we conducted pre- and post- evaluations of HRQoL and perceived social support in order to measure the impact of a nursing student intervention on a rural population in Ecuador.

## Methods

### Intervention

Nursing students at Pontificia Universidad Católica del Ecuador (PUCE) complete the Family Nursing rotation (Fundamentals of Family and Community Nursing) during their sixth semester of study. The primary objective of the rural family service learning experience is to train the nursing students to be able to provide comprehensive care in the community through a combination of clinical intervention, health promotion and education, and linkages with the local public health facilities. The intervention was provided only to the experimental community and was supervised by nursing faculty at PUCE. Faculty at PUCE provided the students with a checklist of interventions from the Nursing Intervention Classification (NIC) to be administered to each family that were appropriate to the students’ level of competency and in-line with the course objectives. The checklist included 63 intervention activities from 10 of the 30 NIC classes: Self-Care Assistance, Exercise Promotion, Active Listening, Coping Enhancement, Self-Esteem Enhancement, Family Support, Socialization Enhancement, Support System Enhancement, Risk Identification, and Health Education [30]. The students were also instructed to develop customized plans of care according to the individual family’s needs.

Each nursing student was assigned two families in the experimental community to visit each week for 8 weeks. At the initial home visit, the students conducted a needs assessment and developed a plan of care for the family. At each subsequent home visit, the students carried out specific objectives relating to the plan of care, including vital sign monitoring, educational talks, chronic disease management support, and basic clinical interventions. The students provided the families with health promotion materials, basic medical supplies, and customized nutrition plans. When necessary, the nursing students made referrals to local public health facilities and/or communicated directly with the family’s primary care provider to ensure continuation of care. While all nursing students were expected to complete the checklist of NIC items for each family, the remaining aspects of the intervention varied widely according to the families’ needs. Examples of interventions provided included diabetes management, prenatal care, childhood immunizations, sexual health education, and mobility exercises.

### Study design and setting

This study is a quasi-experimental non-equivalent control group design (NCGD). Research partners at the Universidad San Francisco de Quito identified two rural communities in Tumbaco, Ecuador, to receive the intervention over the course of the academic semester. Tumbaco is a rural parish (*parroquia*) located within the Metropolitan District of Quito (DMQ).

Rural populations in the DMQ are fitting candidates for community health interventions, as the area is experiencing an increasing trend of private health facilities and a reduction in public facilities accessible to the poor [31]. The community receiving the intervention during the course of this study was designated as the experimental group, while the community scheduled to receive the intervention in the following semester was designated as the control group. Because assignment to the experimental group was not random, the researchers chose a control community similar in size and containing a population of comparable family type, age, and socioeconomic status, as determined by statistical tests for independence (see Results). Though the intervention was delivered at the household level, the unit of analysis for this evaluation is the individual study participant, as HRQoL and perceived social support are concepts measured at the individual level. Thus, individual members of families receiving the intervention were selected for the study.

Members of both communities were invited to participate in the study through a door-to-door recruitment process in which each community was divided into sectors, and a data collection team made up of one public health researcher and one nursing student was assigned to each sector. Each team attempted to approach every home in the sector during days of the week when most families would be at home in an effort to recruit one member of each household into the study. Eligibility criteria for study participation included the ability to read and write in Spanish and an age of 18 years or older. Families or individuals who had participated in similar interventions within the last 18 months were excluded. Informed and written consent was obtained for all study participants, and all study procedures were approved by the institutional review boards at the Universidad San Francisco de Quito and the University of Georgia.

### Data collection and instruments

Pre-intervention data collection occurred in June and July 2015 for both the experimental and control groups. Four surveys were administered to participants at this time: 1) a family health questionnaire; 2) a measure of socioeconomic status; 3) a HRQoL survey; and 4) a measure of perceived social support. Post-intervention data collection occurred December 2015 – January 2016 in both groups and included the HRQoL survey and the measure of perceived social support.

The family health questionnaire was developed by a team of researchers at PUCE and served as a needs assessment for the nursing students as they developed their plan of care for the family. It contained questions on demographic information, health behaviors, and healthcare utilization. This questionnaire has been used in multiple communities throughout Ecuador [32].

Socioeconomic status was derived using a questionnaire developed by Ecuador’s Instituto Nacional de Estadística y Censos [33]. The INEC questionnaire obtained information about the family’s living conditions, access to technology, consumer behavior, and level of education and translated these answers into a score between 0 and 1000. This score then corresponded to one of five socioeconomic groups, labeled “Low” to “High”.

HRQoL was measured at the individual level using version two of the 12-item short-form health survey (SF-12v2). The SF-12v2 has demonstrated adequate validity and reliability in the United States and internationally [34–36], and the Spanish language version has been used successfully in Latin America and with Spanish-speaking populations in the United States [37–40]. The survey measures HRQoL across eight health domains: physical function (PF), physical role (RP), bodily pain (BP), general health (GH), vitality (VT), social function (SF), emotional role (RE), and mental health (MH). Domain scores were used to calculate two component summary scores: the physical component summary (PCS) and the mental component summary (MCS). Questionnaires were scored and raw scores were transformed to a norm-based 0–100 scale according to Quality Metric guidelines [41]. The final norm-based scores correspond to a mean of 50 and a standard deviation of 10, meaning that scores above 50 are higher than the average score of the reference population. For this study, a 1998 general United States population was used as the reference population because population coefficients for Ecuador were not available, and US-derived scores are the recommended international standard for comparison [35].

Perceived social support was measured at the individual level with the 12-item short-form Interpersonal Support Evaluation List (ISEL-12) [23]. The ISEL-12 comprises a series of Likert scale items, and scoring the instrument involved summing all of the items to obtain an overall score of perceived social support. Individual items are designated as part of one of three social support domains that may be scored to provide corresponding subscale scores: appraisal (instructional support/advice), belonging (emotional support/acceptance), and tangible (material support/assistance). Scores range from 0 to 36, but there is no general population coefficient available for norm-based scoring. The Spanish language version of the ISEL-12 has been validated and used among Latino populations in the United States and in Latin America [42–44].

### Study sample

**Fig. 1:**
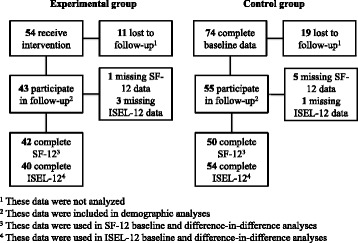
Flow diagram of experimental and control groups. Flow diagram indicating the number of participants in each group (experimental and control) at baseline, the number lost to follow-up, the number participating in follow-up surveys, and the final study sample for each group

Members of 54 households in the experimental community were enrolled in the intervention, and 74 households in the control community participated in baseline data collection. Eleven intervention participants (20.4%) and 19 control participants (25.7%) were lost to follow-up. Additionally, one intervention participant and five control participants were missing SF-12 data and were excluded from the SF-12 analysis, while three intervention participants and one control participant were missing ISEL-12 data and were excluded from the ISEL-12 analysis. The final sample included 43 intervention participants and 55 control participants, with 42 intervention and 50 control participants in the SF-12 analysis and 40 intervention and 54 control participants in the ISEL-12 analysis. Figure 1 summarizes the study sample.

### Data analysis

Data were analyzed using R Statistics version 3.3.3 [45]. To test the independence of the experimental and control communities, Chi-square tests were used for categorical variables and Wilcoxon Mann-Whitney tests were used to compare continuous demographic variables. Wilcoxon Mann-Whitney tests were also used to test for significant differences in SF-12 and ISEL-12 scores between groups at baseline, as nonparametric testing was recommended due to the skewedness of the data and the small sample size.

The impact of the nursing intervention was estimated using difference-in-differences analysis, which compared the change in SF-12 and ISEL-12 scores from baseline to post-test for the experimental group to the changes in scores for the control community. We chose this method of analysis in order to mitigate the threat to internal validity incurred through non-randomization and our inability to control for many exogenous factors due to limited data collection. Scores could not be assumed to follow a normal distribution; therefore Wilcoxon Mann-Whitney tests were used to perform the difference-in-differences analysis.

## Results

### Participant characteristics

A demographic comparison of the two study groups is summarized in Table 1. The average age in the experimental group was 45.9 years, with 79.1% female, compared to 37 years and 63.6% female in the control group. The average number of family members in an experimental household was 4.1, and the mean socioeconomic score was 510.4. The control households had an average of 4.5 family members, and the mean socioeconomic score was 524.1. Wilcoxon Mann-Whitney and Chi-square tests for independence showed no significant differences between groups for the percent female, number of family members, or socioeconomic score. The experimental group participants were significantly older than the control group participants; however, a measure of Spearman’s rank correlation between age and change in SF-12 and ISEL-12 scores showed no significant correlation between age and score change for any domain score or composite score (all *p*-values > 0.05).

**Table 1:** Demographic variables of experimental and control communities

Variable	Experimental	Control	*p*-value
Age, Mean (SD)	45.9 (18.3)	37.0 (16.3)	.015
Female, Number (%)	34 (79.1)	35 (63.6)	.150
Family size, Mean (SD)	4.1 (2.4)	4.5 (1.8)	.119
INEC, Mean (SD)	510.4 (157.2)	524.1 (142.2)	.689

### Baseline scores

Table 2 reports the baseline SF-12 and ISEL-12 scores for both the experimental and control groups, along with the *p*-value results of the Wilcoxon Mann-Whitney tests for all domain and composite scores. Mean scores for every SF-12 domain except Vitality are below 50, indicating that the average HRQoL for this population is lower than the average score of the reference population. There are no significant differences between groups at baseline for any composite summary scores or any of the SF-12 domains.

**Table 2:** Baseline scores of experimental and control communities, mean (SD)

Variable	Experimental	Control	*p*-value
SF-12 PCS	41.1 (9.7)	42.1 (9.1)	.678
PF	40.5 (12.2)	43.2 (10.3)	.255
RP	39.0 (10.8)	39.9 (9.0)	.831
BP	42.4 (10.9)	42.2 (11.7)	.974
GH	39.6 (11.3)	39.7 (11.9)	.934
SF-12 MCS	44.8 (9.1)	45.0 (11.4)	.975
VT	56.6 (10.9)	56.0 (9.2)	.583
SF	40.2 (13.6)	40.4 (12.9)	.971
RE	35.2 (11.1)	37.0 (11.9)	.535
MH	45.7 (9.5)	46.1 (11.3)	.638
ISEL-12 Total	20.7 (4.9)	22.3 (4.4)	.074

Legend Abbreviations: *PCS* Physical Component Summary, *PF* physical function, *RP* physical role, *BP* bodily pain, *GH* general health, *VT* vitality, *SF* social function, *RE* emotional role, *MH* mental health, *MCS* Mental Component Summary, *ISEL* Interpersonal Support Evaluation List

### Effect of intervention

**Fig. 2:**
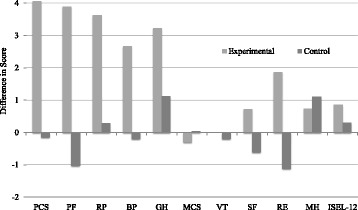
Average difference in summary measures for experimental and control groups. Bar chart depicting the average difference in each summary measure for each study group, from pre to post. Average score differences for the experimental and control groups are displayed side-by-side, including the two composite and eight domain scores of the SF-12v2, as well as the ISEL-12 scores. Legend Abbreviations: *PCS* Physical Component Summary; *PF* physical function; *RP* physical role; *BP* bodily pain; *GH* general health; *VT* vitality; *SF* social function; *RE* emotional role; *MH* mental health; *MCS* Mental Component Summary; *ISEL* Interpersonal Support Evaluation List

Figure 2 displays the average difference in each summary measure for the experimental and control groups. The intervention group realized mean score improvement across all domains and composite scores except for the mental component summary (MCS) score of the SF-12. The control group showed less improvement between pre- and post- testing for the majority of measures, with average decreases in pre to post for five summary measures.

Table 3 reports the results of the difference-in-differences analysis for the two communities. When compared to the control group, the experimental group realized significant improvements in the physical component summary (PCS) score of the SF-12 and the physical function (PF) domain of the SF-12. There were no statistically significant differences for any other components of the SF-12 or ISEL-12.

**Table 3:** Difference-in-differences analysis

Measure	DD	*p*-value	Sig^a^
SF-12 PCS	4.20	0.032	^b^
PF	4.92	0.043	^b^
RP	3.34	0.220	
BP	2.87	0.235	
GH	2.11	0.185	
SF-12 MCS	−0.34	0.464	
VT	0.20	0.918	
SF	1.33	0.813	
RE	2.98	0.780	
MH	−0.37	0.717	
ISEL-12	0.55	0.495	

^a^Significance level: ^b^ = 5%

## Discussion

The aim of this study was to determine the impact of a healthcare intervention conducted by nursing students on the health-related quality of life (HRQoL) and perceived social support of a rural population in Ecuador. To our knowledge this is the first study to examine the impact of nursing student service provision on the population served instead of on the student providers in a low- or middle-income country. Overall, this analysis demonstrated that students completing their rural Family Nursing rotation have the potential to improve the health-related quality of life and perceived social support of rural residents in Ecuador. The statistically significant impact of the intervention was limited to small improvements in the physical function domain of the SF-12v2 and the overall physical component summary score of the SF-12v2, but mean improvements across nearly every health-related quality of life and interpersonal support measure were realized for the experimental group.

Additionally, this study introduces HRQoL as an outcome measure for health sciences students completing their LOES community internship requirements. More traditional measures, such as body mass index or blood pressure, may not be appropriate target outcomes for an 8-week student intervention, as significant changes in these measures are difficult to achieve in such a short time period. HRQoL is more amenable to short-term interventions and, if assessed repeatedly and in a variety of settings, could serve as an indicator of the success of Ecuador’s LOES community improvement objective as well as other service-based learning programs.

### Limitations

Certain limitations should be considered when examining the results of this study. First, the study participants were not randomized into experimental and control groups; randomization into study group was not feasible, as the intervention administrators lacked the capacity to deliver the intervention simultaneously across multiple communities. One consequence of this non-randomization is the higher mean age of the experimental group. Although HRQoL is often associated with age [35], the *change* in HRQoL from pre- to post-intervention was not found to be significantly correlated with age in this sample. Difference in differences analysis was chosen in an effort to mitigate the effects of non-randomization, but the potential for selection bias remains. Second, the decision to limit study eligibility to participants who could read and write in Spanish mitigated the potential effect of interviewer bias at the expense of introducing selection bias, as those in the study area who could not meet the literacy requirements were excluded from participation in the surveys. Illiterate members of participating households were not precluded from receiving the intervention, but their exclusion from the surveys is likely to have reduced the generalizability of our results. Third, the study sample was small, and many were lost to follow-up after baseline data collection due to participants leaving the community, a work schedule that precluded them from participating in the intervention, or being otherwise unavailable during follow-up data collection. Similarly high rates of loss to follow-up are not uncommon in low resource settings [15, 16, 21]. Despite the small sample size, however, statistically significant differences between experimental and control conditions existed, suggesting that effects could have been even greater if the study was better powered.

Additionally, the use of a 1998 United States population as the reference group for the norm-based scoring of the SF-12v2 limits the conclusions that can be drawn about the study sample’s scores relative to other populations. The baseline SF-12v2 scores for this sample were below average for nine of ten summary measures, but it is unclear whether this actually signifies a lower quality of life or is instead a factor of inappropriate population weights. Despite this limitation in comparisons with other populations, use of the US population weights did not limit the conclusions drawn from differences between pre and post for our experimental and control groups.

Finally, the intervention was not fully standardized across the experimental group. The nursing students were required to complete a checklist of NIC activities for each family, but the remaining aspects of the intervention were customized to each family’s needs. Measuring the impact of an intervention that provides prenatal care and childhood immunizations to one family and chronic disease management to members of another precludes a measurement tool that is precise and narrow in scope. Instead, a broad intervention requires a general measure; thus, we chose the SF-12v2 and the ISEL-12 in an effort to capture general improvements in our experimental population, but it is possible that the chosen measurement tools were not equipped to recognize additional manners in which the intervention may have improved the quality of life and wellbeing of the participants.

## Conclusion

The results of this study are promising regarding the potential for health sciences students in a service-based learning course to contribute to improved health outcomes in a rural population. This study fills a gap in the literature by examining the impact of nursing student service provision on the HRQoL and perceived social support of the population served. Additionally, this study contributes to the evaluation of Ecuador’s LOES community improvement objective by introducing HRQoL as a potential indicator of health sciences students’ success in service-based learning programs. Further studies of this kind are necessary to determine if similarly positive findings are realized for other rural populations in Ecuador and globally.
